# Vascular calcification is coupled with phenotypic conversion of vascular smooth muscle cells through Klf5-mediated transactivation of the Runx2 promoter

**DOI:** 10.1042/BSR20140103

**Published:** 2014-11-04

**Authors:** Jing Zhang, Bin Zheng, Pei-pei Zhou, Ruo-Nan Zhang, Ming He, Zhan Yang, Jin-Kun Wen

**Affiliations:** *Department of Biochemistry and Molecular Biology, the Key Laboratory of Neural and Vascular Biology, China Administration of Education, Hebei Medical University, No. 361, Zhongshan East Road, Shijiazhuang 050017, China; †The fourth hospital of Shijiazhuang, Shijiazhuang 050011, China

**Keywords:** high phosphate, Klf5, *Runx2*, vascular calcification, vascular smooth muscle cell (VSMC), Ad-Klf5, adenovirus carrying the Klf5 gene, Ang II, angiotensin II, ChIP, chromatin immunoprecipitation, CRF, chronic renal failure, DMEM, Dulbecco’s modified Eagle’s medium, GAPDH, glyceraldehyde-3-phosphate dehydrogenase, GFP, green fluorescent protein, Klf5, Krüppel-like factor 5, KLFs, KLF family, Pi, phosphate, siRNA, small interfering RNA, SM α-actin, smooth muscle α-actin, SM22 α, smooth muscle 22 α, SRF, serum-response factor, VSMC, vascular smooth muscle cell, TCE, transforming growth factor-β control element

## Abstract

Both Klf5 (Krüppel-like factor 5) and Runx2 are involved in phenotypic switching of VSMC (vascular smooth muscle cells). However, the potential link between Klf5 and Runx2 in mediating vascular calcification remains unclear. The aim of the present study was to elucidate the actual relationship between Klf5 and Runx2 in mediating VSMC calcification. We found that high Pi (phosphate) increased the expression of Klf5, which is accompanied by loss of SM α-actin and SM22α (smooth muscle 22 α), as well as gain of *Runx2* expression. Overexpression of Klf5 increased, while knockdown of Klf5 decreased, *Runx2* expression and calcification. Further study showed that Klf5 bound directly to the *Runx2* promoter and activated its transcription. Klf5 was also induced markedly in the calcified aorta of adenine-induced uremic rats. In conclusion, we demonstrate a critical role for Klf5-mediated induction of Runx2 in high Pi -induced VSMC calcification.

## Abstract 2

High phosphate induces the expression of Klf5 and VSMC calcification. Klf5 binds directly to the *Runx2* promoter and activates its transcription. Vascular calcification is coupled with phenotype conversion of VSMCs through Klf5-mediated transactivation of *Runx2* promoter.

## INTRODUCTION

Vascular calcification is one of the major complications of atherosclerosis, diabetes mellitus, hypertension and chronic kidney disease. It decreases vessel elasticity and compliance of the vessel wall [1], leading to increased incidence of many cardiovascular diseases, such as myocardial infarction, hypertension and increased plaque rupture during angioplasty [2]. Many actors, including abnormal mineral metabolism, chronic inflammation, and oxidative stress, are associated with the generation of vascular calcification. Accumulating evidence indicates that vascular calcification is not due to passive precipitation of calcium Pi (phosphate), but rather is a tightly regulated process that resembles the ossification processes of the bone [3]. The process includes phenotypic conversion of VSMCs (vascular smooth muscle cells) to a phenotype with osteogenic characteristics. In response to high Pi stimulus, VSMCs increase expression a number of bone-associated proteins such as alkaline phosphatase, MGP, osteopontin and osteocalcin *in vitro* and *in vivo* [4,5], and decrease expression of VSMC differentiation marker genes, including SM (smooth muscle) α-actin and SM22α (smooth muscle 22α) [4].

Recent studies have shown that Runx2, a key transcription factor that regulates osteoblast [6] and chondrocyte differentiation [7], is expressed in atherosclerotic calcified human vascular tissues [6,8] and in calcifying aortic SM cells in mice [9] but not in normal vessels. Runx2 deficiency inhibits oxidative stress-reduced expression of VSMC marker genes and decreases formation of osteoclast-like cells in the calcified lesions [9]. Ang II (angiotensin II) exacerbates the vascular calcification through activation of Runx2 and NF-KB (nuclear factor κB) [10]. Speer et al. showed that Runx2 is required for high Pi- and oxidative stress-induced vascular calcification [11], supporting an essential role of Runx2 in VSMC calcification.

The transcription factors controlling the phenotypic state of VSMCs are also involved in the pathogenesis of vascular calcification. For example, high Pi induces Klf4 (Krüppel-like factor 5) expression in VSMCs. Klf4 knockdown attenuates high Pi-induced SMC (smooth muscle cell) phenotypic switching into osteogenic cells, as well as reduces expression of osteogenic genes and calcium deposition [12]. Klf4 is also induced markedly in the calcified aorta of adenine-induced uremic rats [12]. Our previous studies showed that Ang II inhibits p21 expression by inducing the expression of Klf5, a pro-proliferative transcription factor, and promotes phenotypic conversion of VSMCs into proliferative and synthetic cells [13,14]. KLF4 and KLF5 are closely related members of the KLFs of transcription factors. They bind to a similar DNA sequence that has either a CACCC homology or is rich in GC content [15]. However, it is unknown whether Klf5 exerts any effect on calcification of VSMCs. In addition, the potential link between Klf5 and Runx2-mediated vascular calcification has not been examined. The aims of the present study were to elucidate the effect of Klf5 on VSMC calcification *in vitro* and *in vivo* and to investigate the actual relationship between Klf5 and Runx2 in mediating vascular calcification.

## MATERIALS AND METHODS

### Ethics statement

The investigation conformed to the Guide for the Care and Use of Laboratory Animals published by the United States National Institutes of Health (NIH publication no. 85-23, revised 1996). Approval was granted by the Hebei Medical University Ethics Review Board.

### Cell culture and cell calcification model

VSMCs were isolated from the thoracic aorta of Male Sprague-Dawley rats (80–100 g) as described previously [13]. VSMCs were cultured in DMEM (Dulbecco's modified Eagle's medium) (Gibco) with 10% (v/v)FBS and maintained in 5% (v/v) CO_2_ at 37°C in a humidified atmosphere. VSMCs at passage 3 to 6 were used for all experiments. To induce calcification, after reaching 80% confluence, VSMCs were induced by incubation in calcifying media containing 3.8 mM Pi for 12 days with medium changes every 2 days [16].

### Quantification of VSMC calcification

VSMCs were grown in six-well plates and were treated with the growth medium or calcifying medium. After removing the culture medium and washing with PBS, VSMCs were treated with 0.6 N HCl overnight at 4°C. After removing the HCl supernatant, the cells were washed three times with PBS and solubilized in 0.1 mol/l NaOH/0.1% (w/v) SDS. The calcium content in the HCl supernatant was assayed spectrophotometrically with cresolphthalein. Protein expression was normalized to total cellular protein by the Bradford protein assay.

### Alizarin red S staining

Cells in six-well plates were washed three times with PBS and fixed with 10% (v/v) formaldehyde for 10 min. After three washes with PBS, cells were exposed to 1% (w/v) Alizarin red S for 30 min and washed with 0.2% (v/v) acetic acid. Positively stained cells showed a reddish/purple color.

### Real-time quantitative PCR

Total RNA was isolated with Trizol reagent (Invitrogen) according to the manufacturer's instructions. Reverse transcription-PCR was performed as described previously [14]. The primer sequences for *Klf5*, *Runx2*, ALP (alkaline phosphatase), SM 22 α, SM α-actin and GAPDH (glyceraldehyde-3-phosphate dehydrogenase) were described previously [12,14,17]. The relative expression level was determined using the 2^−ΔΔCt^ method.

### Western blotting

Western blotting was performed as described previously [18]. Antibodies used were as follows: Runx2 (Novus), Klf5 (GeneTex), SM22 α (Abcam), SM α-actin (Epitomics) and GAPDH (Santa Cruz).

### siRNA (small interfering RNA) transfection

siRNA against rat Klf5 and negative control siRNA were designed and synthesized by GenePharma Company (Shanghai). The siRNA sequences for Klf5 were 5′- GCAGACCUAACUUCAUAUATT (sence) and 5′- UAUAUGAAGUUAGGUCUGCTT -3′ (antisence); transfection was performed using Lipofectamine reagent (Invitrogen) following the manufacturer's instructions.

### Luciferase assay

NIH-3T3 cells were transfected with promoter reporter plasmids or the control reporter plasmid pRL-TK using Lipofectamine 2000 reagent (Invitrogen) according to the manufacturer's instructions. 24 h after transfection, cells were treated with high Pi (3.8 mM) for 24 h. Cells were then harvested and luciferase assays were performed using a dual-luciferase assay kit (Promega). Specific promoter activity was expressed as the relative activity ratio of firefly luciferase to Renilla luciferase. All promoter constructs were evaluated in ≥3 separate wells per experiment.

### Quantitative ChIP (chromatin immunoprecipitation) assays

The ChIP assay was carried out as described in the ChIP Assay Kit (Millipore) instructions. Briefly, VSMCs were treated with 1% (v/v) formaldehyde for 10 min to cross-link proteins with DNA. The cross-linked chromatin was then prepared and sonicated to an average size of 400–600 bp. The DNA fragments were immunoprecipitated overnight with anti-KLF5 antibody. After reversal of cross-linking, the genomic region of Runx2 flanking for Klf5 binding sites were amplified by PCR with the following primer pairs: P1-F, 5′-CTATTTTGACATGCCCTCCTG-3′; P1-R, 5′-GTCCGAGTTGACTTCTGAGC-3′; P2-F, 5′-GC-CTTAGCTTGGGTCGTGTC-3′; P2-R 5′-CAAAAGGTTGT-GGTTGAGAGATG-3′; P3-F, 5′-CCGATATTGCTTCTGCC-TAGTTC-3′; P3-R 5′-GATCATTATGCTGATGGGAGTCAG-3′; P4-F, 5′-GGATCCTGACAGGTCTCTTGC-3′; P4-R, 5′-TGACACAACTGGGATTTGGTG-3′.

### Oligonucleotide pull-down assay

The oligonucleotide pull-down assay was carried out as described previously [19]. Oligonucleotides containing the TCE (transforming growth factor-β control element) site (Site 1-2) sequence in the rat Runx2 promoter with biotin added to their 5′-end were as follows: Biotin-5′-CTCCTGT-CACCCTTTTCTGATGTCACCTAAGGGGGGAAAAACCA-CCCCCGAA -3′ (forward) and Biotin-5′- TTCGGGGGT-GGTTTTTCCCCCCTTAGGTGACATCAGAAAAGGGTGA-CAGGAG-3′ (reverse) for site 3-4; Biotin-5′-CCCCCCCGC-CCCAGCCAATGTGGGGTGGGGTTGGGGGGGAAGGGT-GAAGA (forward) and Biotin-5′-TCTTCACCCTTCCC-CCCCAACCCCACCCCACATTGGCTGGGGCGGGGGGG (reverse) for site-3-4, Each pair of oligonucleotides was annealed following standard protocols.

Cell extracts were precleared with ImmunoPure streptavidin-agarose beads (20 μl sample, Pierce) and then incubated with 100 pmol of biotinylated double-stranded oligonucleotides and 10 μg of poly (deoxyinosine:deoxycytidine). DNA-bound proteins were captured with 30 μl of immobilized streptavidin-agarose beads followed by extensive washing. Bound proteins were separated by SDS–PAGE and subjected to Western blotting.

### Adenine-induced CRF (chronic renal failure) rats

The CRF animal model was established as described previously [20]. Eight-week-old male Wistar rats were fed a standard diet containing 1.2% calcium and 0.99% phosphorus alone or a diet containing 0.75% adenine for 6 weeks to induce uremia. After 6 weeks, the rats were anesthetized with chloral hydrate, and blood was collected to measure serum creatinine, urea nitrogen, calcium and Pi et al biochemical parameters. Abdominal aortas were excised and embedded by OCT and Frozen sectioned for von Kossa staining and morphological analysis. For measurement of calcium content, the tissue sample was weighed and then hydrolysed in 0.5 ml of 0.6N HCl for 24 h and then determined using a commercially available kit as described previously [21]. Aortic extracts were performed real-time PCR and Western blot analysis as described previously.

### Statistical analyses

Data are presented as bar graphs of the mean±S.E.M. of ≥3 independent experiments. Statistical analyses were performed using the Student's *t* test or one-way ANOVA according to the number of groups compared. Differences were considered significant at *P*<0.05.

## RESULTS

### High Pi-induced *Klf5* expression and VSMC calcification

**Figure 1:**
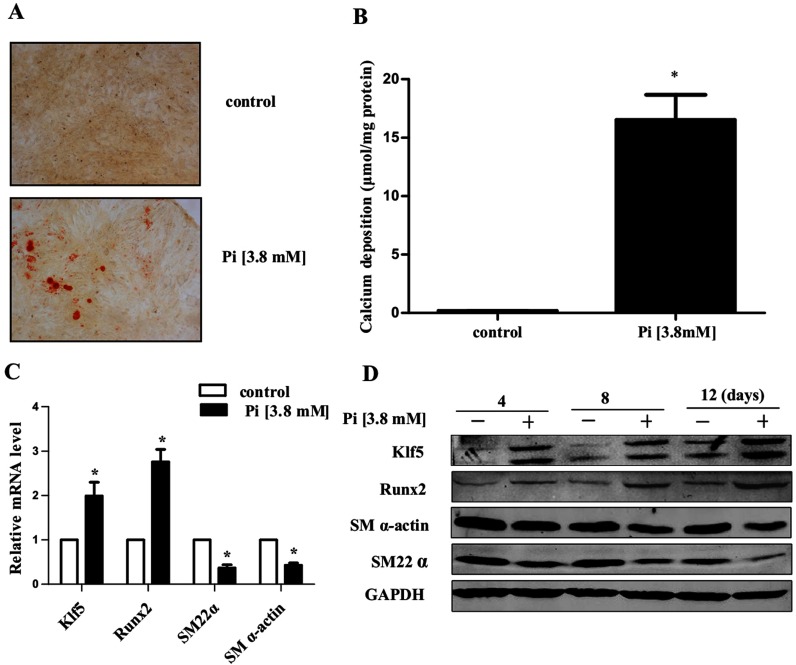
High Pi-induced Klf5 expression and VSMC calcification (**A**) Rat aortic VSMCs were cultured with control or high Pi (3.8 mM) medium for 12 days. Representative pictures of Alizarin red S staining are shown (*n*=3). Scale bar, 20 μm. (**B**) Quantitative analysis of calcium deposition. VSMCs cultured in control or high Pi medium for 12 days. VSMCs were lysed with 0.6N HCl overnight. Total calcium in the lysates was determined by O-cresolphthalein method and normalized by protein concentration. Values are expressed as means±S.E.M. from three independent experiments. *P<0.05 compared with VSMCs with control medium (*n*=3). (**C**) VSMCs were incubated with control or high Pi medium for 6 days. Expression of *Klf5*, *Runx2*, *SM* α*-actin* and *SM22*α mRNA was determined by real-time PCR. Values represent the means±S.E.M.. *P<0.05 compared with VSMCs incubated with control medium (*n*=3). (**D**) Expression of *Runx2*, *Klf5*, *SM α*-actin and *SM22α* in VSMCs incubated with control or high Pi medium for 4, 8 and 12 days was examined by Western blotting.

Previous studies have shown that the high Pi concentration induced VSMC calcification through increasing *Runx2* expression [12]. To investigate the molecular mechanisms whereby high Pi-induced Runx2 expression, we first examined the effect of high Pi on pro-proliferation factor Klf5. We cultured VSMCs for 12 days in control or high (3.8 mM) Pi DMEM with 10% FBS, and the calcification status was determined by Alizarin red S staining. As shown in Figure 1(A), incubation of VSMCs with high Pi medium markedly increased calcification of cultured VSMCs, and total calcium in the cell lysates was enhanced ~100-fold (Figure 1B). Expression of VSMC differentiation- and proliferation-related genes as well as osteogenic gene was examined by real-time PCR and Western blotting. The results showed that incubation with the high Pi medium for 6 days increased Klf5 and Runx2 mRNA expression by 1.0-fold and 1.76-fold, respectively. In contrast, expression of VSMC differentiation marker genes, *SM22α* and *SM α*-actin, was significantly decreased by 43 or 38% with high Pi treatment (Figure 1C). Similarly, the protein levels of SM α-actin and SM22α were reduced in a time-dependent manner in VSMCs cultured with high Pi medium for 4, 8 and 12 days. Conversely, the levels of Runx2 and Klf5 time-dependently increased. These results indicate that high Pi promotes the expression of osteogenic gene *Runx2* and VSMC calcification, and that Klf5 may play an important role in mediating *Runx2* expression in VSMCs.

### *Klf5* overexpression increased *Runx2* expression and VSMC calcification

**Figure 2:**
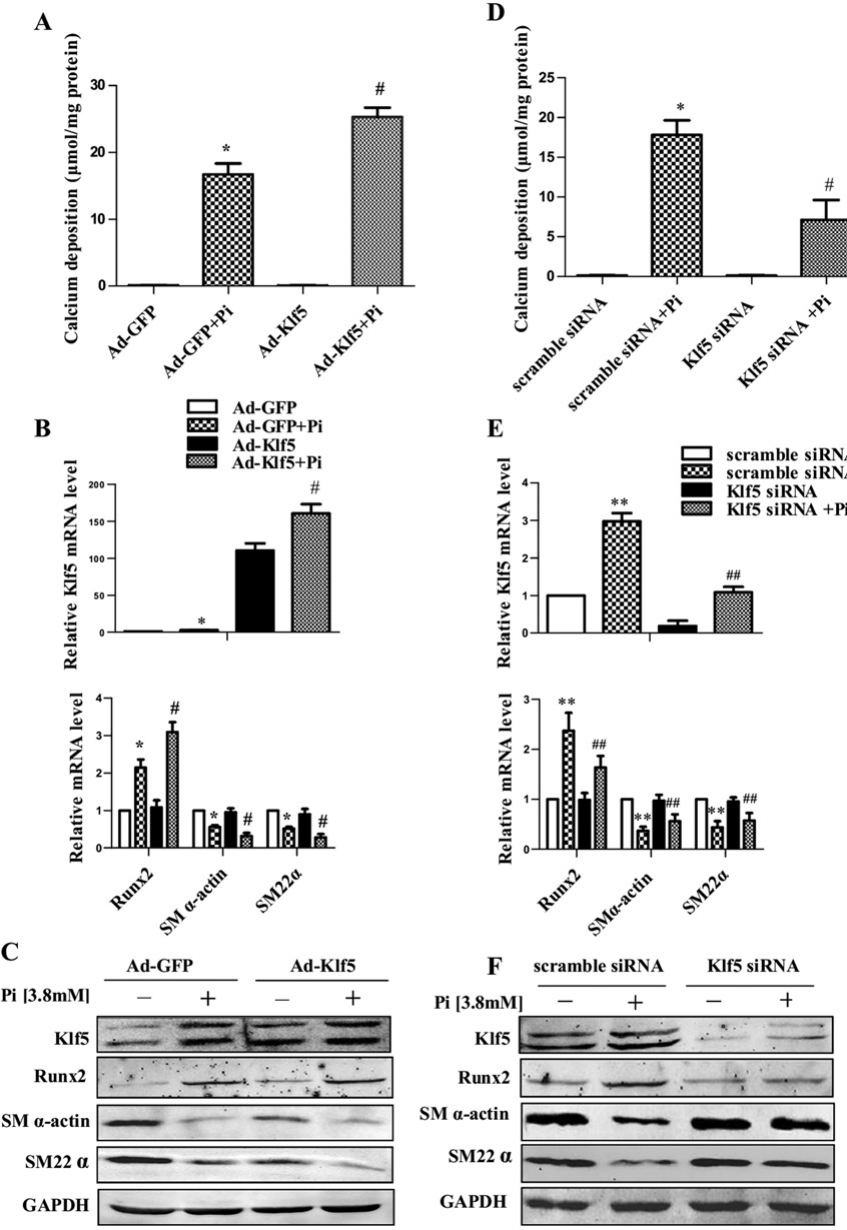
*Klf5*-mediated *Runx2* expression and VSMC calcification (**A**) Quantification of calcium deposition in VSMCs. Rat VSMCs were infected with Ad-GFP or Ad-Klf5 for 48 h and then incubated with high Pi medium for an additional 7 days. (**B**) Relative mRNA levels of *Runx2*, *SM* α*-actin* and *SM22*α in VSMCs overexpressing Klf5 were determined by real-time PCR. (**C**) Runx2, Klf5, SM α-actin and SM22α in VSMCs infected with Ad-Klf5 for 48 h and then incubated with high Pi medium for 7 days were examined by Western blotting (*n*=3). (**D**) Quantification of calcium deposition in VSMCs. Rat VSMCs were infected with scramble siRNA or Klf5 siRNA for 48 h and then incubated with high Pi medium for an additional 7 days. (**E**) Relative mRNA levels of *Runx2*, *SM* α*-actin* and *SM22*α in VSMCs knocking down Klf5 were determined by real-time PCR. (**F**) Runx2, Klf5, SM α-actin and SM22α in VSMCs transfected with scramble siRNA or Klf5 siRNA and then incubated with high Pi medium for 7 days were examined by Western blotting (*n*=3). *P<0.05 versus Ad-GFP. # *P*<0.05 versus Ad-GFP+high Pi. **P<0.05 versus scramble siRNA. ##*P* ≤ 0.05 versus scramble siRNA±high Pi.

To further investigate the potential role of Klf5 in high Pi-induced calcification, we overexpressed Klf5 in VSMCs by Ad-Klf5 (adenovirus carrying the *Klf5* gene) and tested whether *Klf5* overexpression alone could drive calcification in the absence of calcifying medium. First, overexpression of *Klf5* and induction of Klf5 by high Pi were verified at both the mRNA and protein levels (Figures 2B and 2C). Next, we found that Klf5 overexpression did not cause detectable calcification in VSMCs cultured with the control medium during 7-day culture period, as indicated by the calcium-deposition levels (Figure 2A). However, when VSMCs were exposed to high Pi medium, *Klf5* overexpression significantly increased calcium deposition by 60% (Figure 2A). Furthermore, expression of osteoblast marker gene *Runx2* increased 2.3-fold in VSMCs infected with Ad-klf5 plus high Pi treatment when compared with that infected with GFP (green fluorescent protein) adenovirus (Figures 2B and 2C). In addition, the expression of VSMC markers SM α-actin and SM22α was decreased by 50% after VSMCs were exposed to high Pi medium for 7 days. Importantly, both the *Klf5* overexpression and high Pi treatment markedly reduced *SM α*-actin and *SM22α* expression by 40%. These results suggest that induction of Runx2 and loss of SMC-specific phenotypes are associated with Klf5 overexpression and high Pi- induced VSMC calcification.

### Knockdown of *Klf5* decreased *Runx2* expression and VSMC calcification

Because overexpression of *Klf5* promoted *Runx2* expression and VSMC calcification, we sought to determine whether knockdown of Klf5 by siRNA could reduce the calcification of VSMCs. To do this, we knocked down Klf5 in VSMCs and used high Pi to induce calcification. As shown in Figure 2(D), Klf5 knockdown greatly reduced high Pi-induced VSMC calcification, as indicated by calcium deposition in VSMCs compared with that transfected with scramble siRNA. We confirmed that upon exposure to high Pi, calcium deposition in VSMCs knocking down Klf5 was decreased to 30% of the cells transfected with scramble siRNA. Additionally, knockdown of Klf5 significantly decreased the expression of osteogenic marker gene *Runx2* by 75% in response to high Pi stimulation, as indicated by real-time PCR (Figure 2E) and Western blotting (Figure 2F). As expected, knockdown of Klf5 and incubation with the high Pi medium increased the expression of SM α-actin and SM22α when compared with the cells transfected with scramble siRNA and then incubated with high Pi. These results again suggest that Klf5 plays an important role in mediating VSMC phenotypic switching into osteogenic cells and calcification.

### Klf5 bound to the Runx2 promoter and activated its transcription

**Figure 3:**
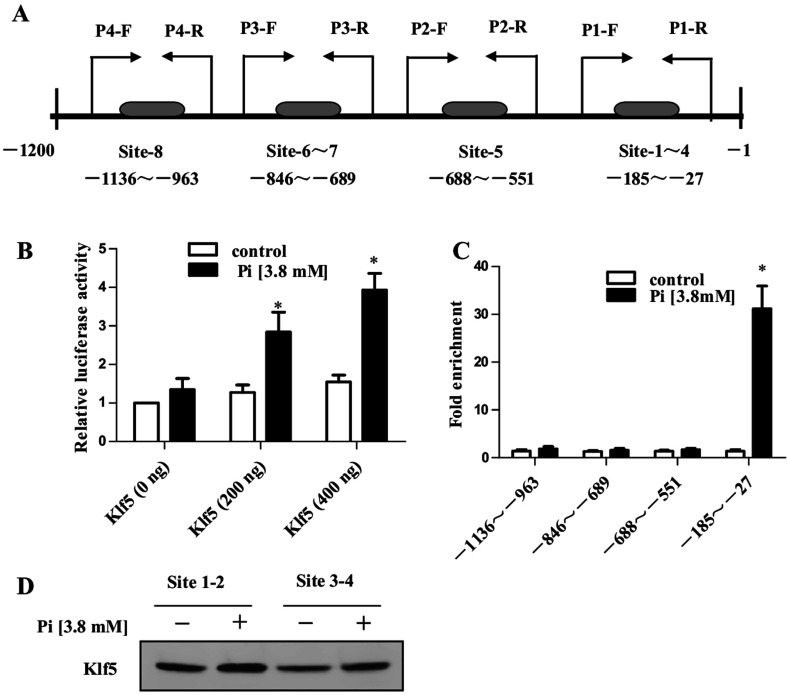
High Pi promoted the binding of Klf5 to the Runx2 promoter (**A**) Schematic map of the Runx2 promoter region-1200 to-1 showing the position of the Klf5-binding sites. The arrows represent the primers for PCR in the CHIP assay. (**B**) NIH-3T3 cells were transfected for 24 h with pEGFP or pEGFP-KLF5 and the *Runx2* promoter–reporter constructs (pGL3–Runx2–Luc), and then they were treated with high Pi (3.8 mM) for 24 h. Cell lysates were subjected to a luciferase activity assay. Data are represented as relative *Runx2* promoter activity normalized to pRL-TK activity. The bars indicate the mean±S.E.M. from three independent experiments. *P<0.05 versus the control group. (**C**) VSMCs were cultured with control or high Pi medium for 6 days, ChIP assay was performed with anti-Klf5 antibody. Immunoprecipitated DNA containing different Klf5-binding sites of the *Runx2* promoter was amplified by PCR. The bars represent the means±S.E.M. from three independent experiments. *P<0.05 versus VSMCs incubated with control medium. (**D**) VSMCs were incubated with or without high Pi medium for 6 days, and the whole-cell lysates were subjected to oligonucleotide pull-down assays with biotinylated double-stranded oligonucleotides containing Klf5-binding site sequences (site-1-4) as probes. DNA-bound proteins were collected with streptavidin-agarose beads and analysed by Western blotting with anti-KLF5 antibody.

As Klf5 overexpression or knockdown affected *Runx2* gene expression, we sought to determine whether Klf5 bound directly to the *Runx2* promoter and activated its transcription. Previous studies have shown that there are several regulatory proteins (such as Msx2, Dlx5, Twists, etc.) play critical roles in modulating *Runx2* gene expression, activity, and the subsequent bone formation [22]. Using the TESS-String-based Search (//www.cbil.upenn.edu/tess/) and AliBaba2/Transfactor 6.0 (//www.iti.cs.uni-magdeburg.de/grabe/alibaba2/) computer programs, we found that the-1200/-1 bp region of the Runx2 promoter contains eight Klf5-binding sites (also known TCE, transforming growth factor-β control element) (Figure 3A). To investigate whether Klf5 activated transcription of *Runx2* gene, we co-transfected VSMCs with the Klf5 expression plasmid and the *Runx2* promoter reporter (pGL3–Runx2–Luc) construct with or without high Pi treatment. Luciferase assay showed that the *Runx2* promoter could be activated by Klf5 overexpression in Klf5 expression plasmid concentration-dependent manner upon high Pi treatment (Figure 3B). To examine whether high Pi-induced Klf5 binding to the *Runx2* promoter, the ChIP assay was carried out, and the Runx2 promoter regions containing TCE sites were amplified with five sets of PCR primers (Figures 3A and 3C). The results showed that high Pi markedly promoted the binding of Klf5 to proximal region of the *Runx2* promoter (-185 bp–27 bp), which contains Klf5-binding sites 1–4;no significant binding of Klf5 was detected when distal *Runx2* promoter region containing Klf5-binding sites 5–8 was amplified. Consistent with the results of the ChIP assays, oligonucleotide pull-down assay showed that the binding of Klf5 to the sites 1–2 and sites 3–4 was significantly increased when cells were treated with the high Pi medium (Figure 3D). These results indicate that high Pi promotes the binding of Klf5 to proximal region of the *Runx2* promoter and increases *Runx2* transcription activation by Klf5 in VSMCs.

### Klf5 was induced in the calcified aorta of uremic rats

**Figure 4:**
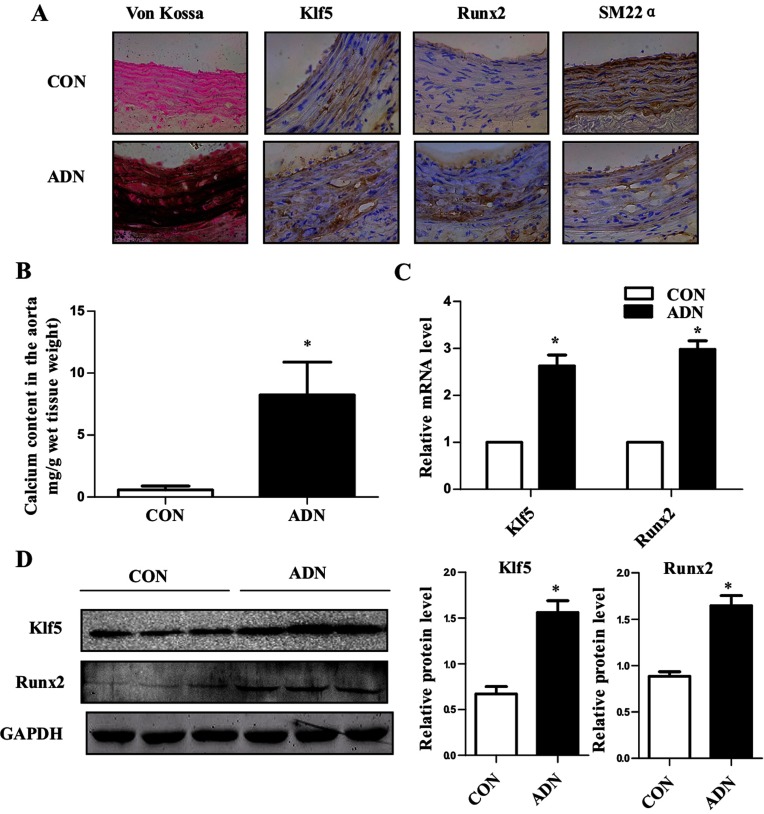
Klf5 was induced in the aorta of adenine-induced uremic rats (**A**) The calcification status and expression of *Runx2*, *Klf5* and *SM22α* were examined by Von Kossa staining and immunohistochemistry in the abdominal aortas of adenine-induced uremic rats and controls. (**B**) Calcium content in the abdominal aorta was expressed as mg/g wet tissue weight. The tissue sample was weighed and then hydrolysed in 0.5 ml of 6N HCl for 24 h. Calcium content was then determined using a commercially available kit. The results were expressed relative to the wet tissue weight. (**C**) *Klf5* and *Runx2* mRNA expression in the aorta of adenine-induced uremic rats and controls was examined by real-time PCR. GAPDH was used as loading control. Data are mean±S.E.M. *P*<0.05 versus control group. (**D**) Representative Western blot analysis and quantification of Runx2 and Klf5. Results are means±S.E.M. normalized to that of GAPDH. *P<0.05 versus control group.

To determine the potential relevance of Klf5 to vascular calcification *in vivo*, we used adenine to induce CRF in rats. Blood biochemistry parameter in CRF rats, including elevated serum creatinine and urea nitrogen, showed that the rat model of CRF was successfully established. By feeding a diet containing 0.75% adenine for 6 weeks, rats exhibited circumferential calcification in the media of abdominal aortas, as indicated by the Von Kossa staining (Figure 4A). Conversely, rats received a control diet showed no significant calcification (Figure 4A). Consistent with the results of the Von Kossa staining, the calcium content in the aorta showed a dramatic increase in adenine-induced uremic rats (Figure 4B). Immunohistochemical staining showed a marked increase in *Klf5* and *Runx2* expression in the aorta of CRF rats compared with control rats, whereas the SM22α expression was reduced in CRF rats (Figure 4A). The expression of Klf5 and Runx2 mRNA and protein was also induced in the aorta of adenine-induced uremic rats, as indicated by real-time PCR (Figure 4C) and Western blotting (Figure 4D). These results further suggest that Klf5 plays a role in VSMC phenotypic switching into osteogenic cells and vascular calcification *in vivo*.

## DISCUSSION

In this study, we identified Klf5 as a key mediator of VSMC osteogenic differentiation and calcification under high Pi conditions *in vitro* and *in vivo*, via direct binding to proximal region of the *Runx2* promoter and increasing *Runx2* transcription in VSMCs.

The high Pi level was found to be important in vascular calcification in clinical trials and *in vitro* and *in vivo* experimental models [17,23]. The potential mechanisms underlying high Pi-induced calcification of VSMCs include osteogenic/chondrogenic conversion of VSMCs, apoptosis, MV (matrix vesicle) release, loss of inhibitor and matrix remodelling [24]. Our study focused on the high Pi-induced VSMC phenotypic switching into osteogenic cells. The results of this study are consistent with the previous reports that high Pi induces calcification and phenotypic conversion of VSMCs that involve the loss of VSMC differentiation markers, such as SM α-actin and SM22α, and the simultaneous gain of osteogenic phenotype, for example, expression of Runx2 [12]. MicroRNAs play a crucial role in diverse biological and pathological processes, including cardiovascular remodelling. Rangrez et al. [25] showed that high Pi treatment causes down-regulation of miR-143 and miR-145 and concomitant up-regulation of their targets and synthetic/activated VSMC markers, such as Klf4 and Klf5. Additionally, miR-145 was identified as part of the specific miRNA profile of destabilized human plaques [26], a biomechanical failure of the plaque that may involve microcalcification [27]. So we speculate that pi controls Klf5 expression by modulating microRNAs.

Interestingly, we found that Klf5 regulated the transcription of the osteogenic transcription factor Runx2 in VSMCs. Runx2 belongs to the runt-related transcription factor family, has been shown to be a key regulator in the expression of several genes involved in bone formation [28]. It is essential for osteoblast differentiation and chondrocyte maturation [6,7]. Multiple signals, including Ang II stimulation, oxidative stress, and high Pi converge on the Runx2 transcription factor to regulate osteoblast differentiation [29]. Indeed, our study showed that the expression of Runx2 was elevated in calcified VSMCs induced by high Pi and vascular tissue from adenine-induced uremic rats, suggesting that Runx2 expression is essential for vascular calcification. In our study, the increase in Runx2 expression was accompanied by a decrease of the expression of VSMC differentiation marker genes SMα-actin and SM22α. A previous study has demonstrated that Runx2 repressed the transcriptional activity of VSMC differentiation marker genes by inhibiting interaction of myocardin with SRF (serum-response factor) and disrupting the formation of the myocardin/SRF complex, which in turn repressed myocardin-mediated expression of the VSMC marker genes [30]. Recently, Yoshida et al. reported that Klf4 contributed to high Pi-induced phenotypic conversion of VSMCs into osteogenic cells through binding to the promoter regions of VSMC differentiation marker genes and suppressing their transcription [12]. They showed that *Runx2* repressed the transcriptional activity of SMC differentiation marker genes by inhibiting myocardin to bind with SRF, and that Runx2 also increased osteopontin mRNA expression in SMCs. Effects of Klf4 and Runx2 are independent of the each other. However, despite these advances, the potential link between the phenotypic conversion of VSMCs and calcification remains unknown.

Klf5 has been shown to have a positive effect on cell-cycle progression and proliferation. Klf5 expression is strongly induced in activated VSMCs in atherosclerosis [31]. The KLFs (KLF family) shares similar zinc finger structures with the Sp family, and some members, such as Sp3 and Sp7 (osterix), are known to be essential for skeletal development and ossification [32,33]. Recently, Kim et al. reported that Klf4 interacted directly with Runx2 promoter and inhibited its expression, attenuated osteoblast formation, function and cross-talk with osteoblast [34]. KLFs are implicated in developmental as well as pathologic vascular processes. In the present work, *in silico* prediction revealed that the Runx2 proximal promoter contains eight binding sites for Klf5. Results of our present studies suggested that Klf5 regulates the transcription of *Runx2* gene in VSMCs, and that high Pi-induced Klf5 binds directly to the *Runx2* promoter and activates its transcription. Klf5-up-regulated Runx2, as a key transcription factor that regulates osteoblast, further plays a role in the regulation of SMC differentiation marker genes and osteogenic genes. In the present study, we provide a novel evidence that the KLFs of transcription factors mediate high Pi-induced conversion of SMCs into osteogenic cells through transactivating *Runx2* gene expression. About the mechanism underlying pi-dependent up-regulation of Runx2 by Klf5, we speculate that high Pi might alter the modification of Klf5 or its interaction with other cofactors, subsequently leading to increase in Klf5 binding to the *Runx2* promoter.

We used adenine-rich diet to induce CRF in rats. Within 6 weeks of feeding 0.75% adenine-rich diet, calcification of the tunica media of the aorta was evident with Von Kossa staining. This type of calcification is independent of lipids and seems to be related to the expression of numerous bone-associated proteins [35]. It is generally accepted that dysregulated Ca and P homoeostasis plays a major role in driving VSMC calcification in CRF. In addition to increasing the Ca×P product, elevated Ca and P can act directly on VSMCs to drive distinct, as well as overlapping, pathways that predispose to calcification [24]. Thus, vascular calcification is a complex biological process governed at partially by Ca×P product [36]. In our study, there was no significant difference between animal model of CRF and control rats in serum calcium, but there was more pronounced effect on serum Pi, which results in elevated Ca×P product and calcification.

In conclusion, we provide novel evidence that Klf5 mediates the high Pi-induced expression of the osteogenic transcription factor Runx2 in VSMCs. Klf5 exerts dual functions in the vascular process: on the one hand, Klf5 promotes VSMC dedifferentiation and prolifetation; on the other hand, it induces the conversion of dedifferentiated VSMCs into osteogenic cells and calcification through transactivating the *Runx2* promoter. Targeting Klf5 or Klf5-regulating signals in VSMCs might represent a novel strategy for prevention and therapy of vascular calcification.
